# Cardiac Lymph Flow Features and New Opportunities for Their Experimental Visualization

**DOI:** 10.1134/S1607672924601318

**Published:** 2025-01-22

**Authors:** P. P. Iablonskii, A. S. Lazareva, I. A. Garapach, A. A. Iablonskaia, S. V. Orlov

**Affiliations:** 1 Laboratory of Microangiopathic Mechanisms of Atherogenesis, St. Petersburg State University, St. Petersburg, Russia; 2 Leningrad Regional Hospital, St. Petersburg, Russia; 3 State Research Institute of Phthisiopulmonology, St. Petersburg, Russia

**Keywords:** lymphatic system, lymphatic vessels of the heart, epicardial lymphatic vessel

## Abstract

The aim of this study was to describe the features of myocardial lymph flow using a new combined method of visualization of the lymphatic system. The study was performed on pig hearts harvested from a local slaughterhouse. The original dye, consisting of lipid-soluble chlorophyll and lipiodol, was injected stepwise into the lymphatic vessels. After sufficient optical identification of the lymphatic vessels, continuous injection of air into the coronary arteries was performed and CT scans were done. In this way, both optical and radiologic visibility of the cardiac lymphatic system was achieved. It was shown that lymph flow of the left and most part of the right ventricle is carried out through lymphatic collectors of the anterior wall of the heart, including retrogradely with respect to the right coronary artery, which complements the previously known facts about the structure of the lymphatic system of the heart.

## INTRODUCTION

The anatomy of the cardiac lymphatic system remains insufficiently studied. Several attempts were made to reveal the anatomy of function of the lymph vessels; however, the regional lymph flow is still underestimated by many clinicians. At the same time, precise knowledge of the localization and physiology of lymphatic vessels could be useful in oncology and cardiovascular surgery, for example, to predict complications of coronary interventions. The cardiac lymphatic system maintains fluid and metabolic balance [1], as well as retrograde transport of cholesterol, which plays a key role in the homeostasis of the vascular wall and the migration of immune cells [2]. It was previously shown that impaired lymphatic transport leads to progressive thickening of the arterial wall in general, as well as the intima–media complex, simultaneously with thinning of the media compared to the contralateral artery in the same individual [3]. Most studies describe the anatomy of lymph in rodents and other small mammals, which differ significantly from humans. Several attempts to map lymphatic vessels on the surface of the pig heart have already been made, although with some limitations [4–8]. Knowledge of the anatomy of the myocardial lymphatic vessels and large arteries could protect us from lymph flow disorders and reduce the incidence of late complications. The aim of this study was to improve and facilitate experimental capabilities of the cardiac lymphatic vessel visualization.

## MATERIALS AND METHODS

In this study, we used pig hearts (*n* = 20) with a fragment of the ascending aorta, pulmonary trunk, and short vein fragments, prepared at a local slaughterhouse immediately after the animals were sacrificed; laboratory animals were not used. The original dye was prepared by the authors from two main components: copper chlorophyll complex diluted in polypropylene glycol in a ratio of 1 : 2 (Copper Chlorophyll Complex, EcoResource, Russia) and lipiodol (Lipiodol Ultra Fluid, Guerbet, France), mixed in a ratio of 1 : 1. This type of dye was chosen because of its lipid solubility, which is necessary to achieve good distribution in lipiodol and lymphatic vessels.

In this study, we focused on the subepicardial lymphatic collectors following the coronary arteries. Therefore, as a first step, to avoid artifacts from myocardial infiltration with the radiopaque agent, pure chlorophyll solution was injected into the myocardium at the apex cordis using a 1-mL insulin syringe and a 26G needle. When the dye reached the lymphatic collectors, the original mixture with lipiodol was injected immediately into the lymphatic vessel using a needle of the same size. Then, the aorta, pulmonary artery, and both atria were hermetically closed with a continuous suture with a polypropylene thread, and an 8F pigtail catheter (PerkuCess HYDRO, Peter Pflugbeil GmbH, Zorneding, Germany) or a similar one was left in the aortic root. During CT scanning, air was continuously injected into the aortic root using an air pump to fill the coronary vessels and ensure negative contrast to the positive contrast provided by lipiodol. CT scanning was performed with a slice thickness of 0.5 mm, a voltage of  80 kV, and a current of 30 mA on a Canon One Aquilon CT scanner (Canon, Tokyo, Japan).

**Fig. 1.**
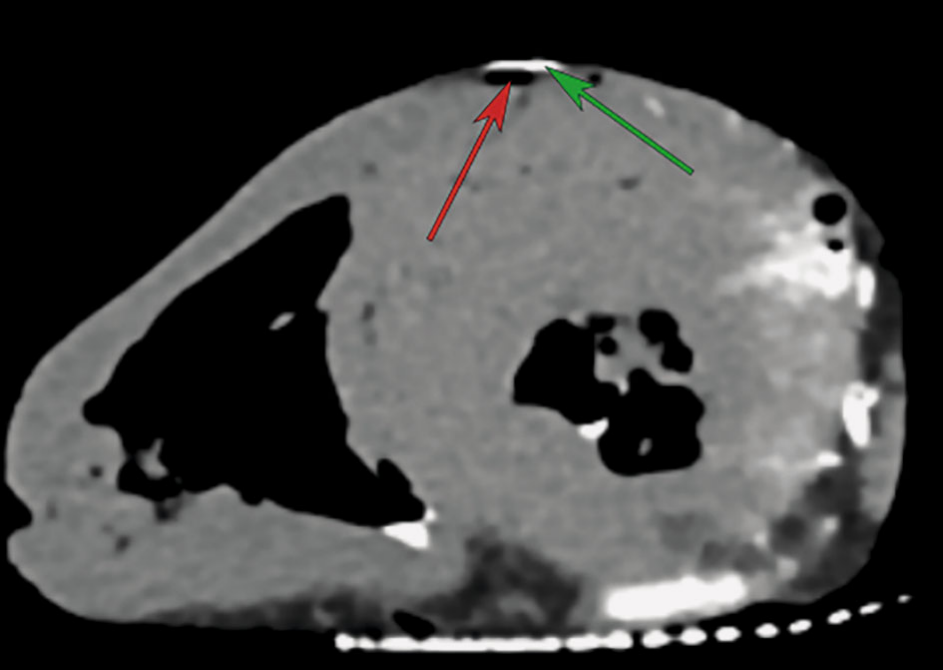
CT scan of a pig heart showing the left anterior descending artery (LAD, red arrow) injected with air and the anterior lymphatic collector (ALC, green arrow) injected with the original dye, the latter forming a bridge over the coronary vessel.

## RESULTS

Visualization of the lymphatic vessels on the anterior wall of the heart was achieved using 2–3 mL of the original mixture. Interestingly, the lymphatic collector was usually located along the coronary artery, but had several “bridges” crossing the left anterior descending artery (LAD). This vessel could be visualized without tissue dissection up to the base of the heart. To a large extent, the anatomy of the lymphatic vessels of the anterior wall in our study was almost identical to the description by Kappler et al. [9]; however, it had several notable features that have not been described previously. The anterior interventricular lymphatic collector showed significant variability in its position along the LAD. Its origin was usually located in the middle of the posterior interventricular artery. Then, it looped around the apex cordis and ran along the LAD, forming the bridges described above. In all cases, the proximal part of the lymphatic vessel was located to the left of the LAD, crossing the artery distally at least twice, in some cases forming multiple (more than three) bridges. In one case, we saw a duplicated vessel running along both sides of the LAD to the midpart of the artery and having several connections. Then, the anterior lymphatic collector ascended almost to the bifurcation point of the left coronary artery trunk, crossed the proximal part of the circumflex artery in a large loop, and went further between the left atrium and truncus pulmonalis to the posterior mediastinum in all cases. Lymphatic drainage of the lateral wall of the left ventricle apparently proceeded in a different way: in this area, a network of small and medium-size lymphatic vessels was observed, which merged with each other into a short trunk only in the atrioventricular groove.

**Fig. 2.**
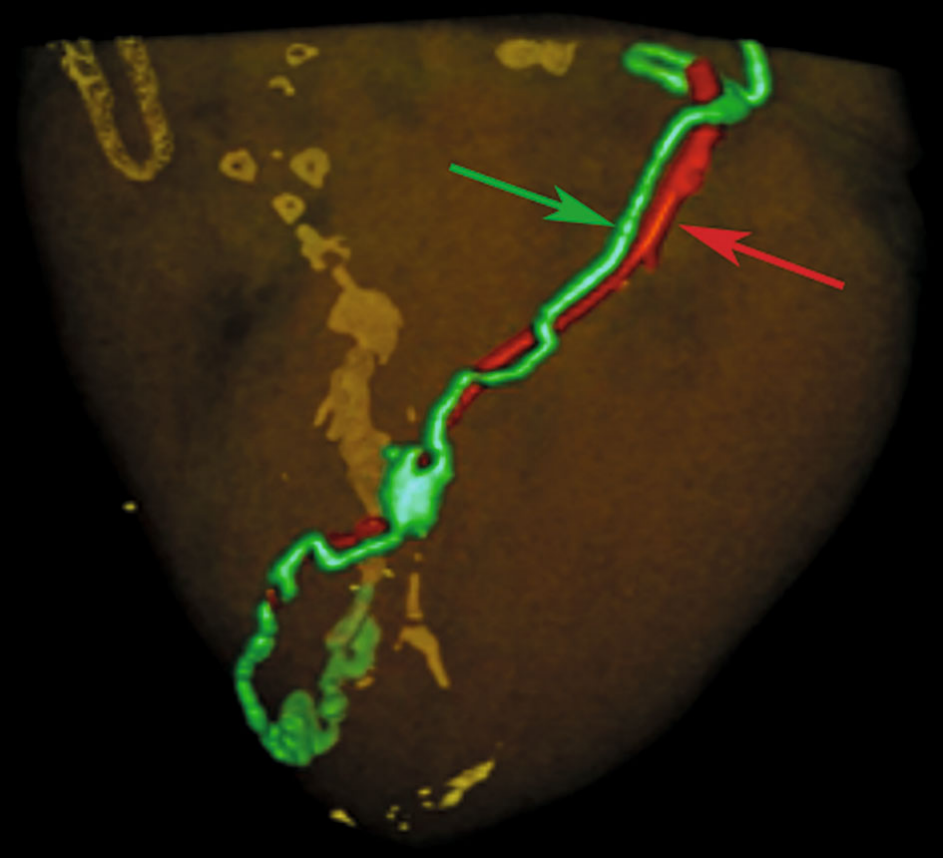
3D reconstructed CT image of the pig heart. The LAD (red arrow) injected with air and the anterior lymphatic collector (ALC, green arrow) injected with the original dye are shown.

The most important observation, from our point of view, is that only the lymph flow from the proximal part of the right coronary artery basin (zones 1 and 2) went through the collector along it and then along the interatrial groove retrocardially, to the confluence with the anterior trunk

**Fig. 3.**
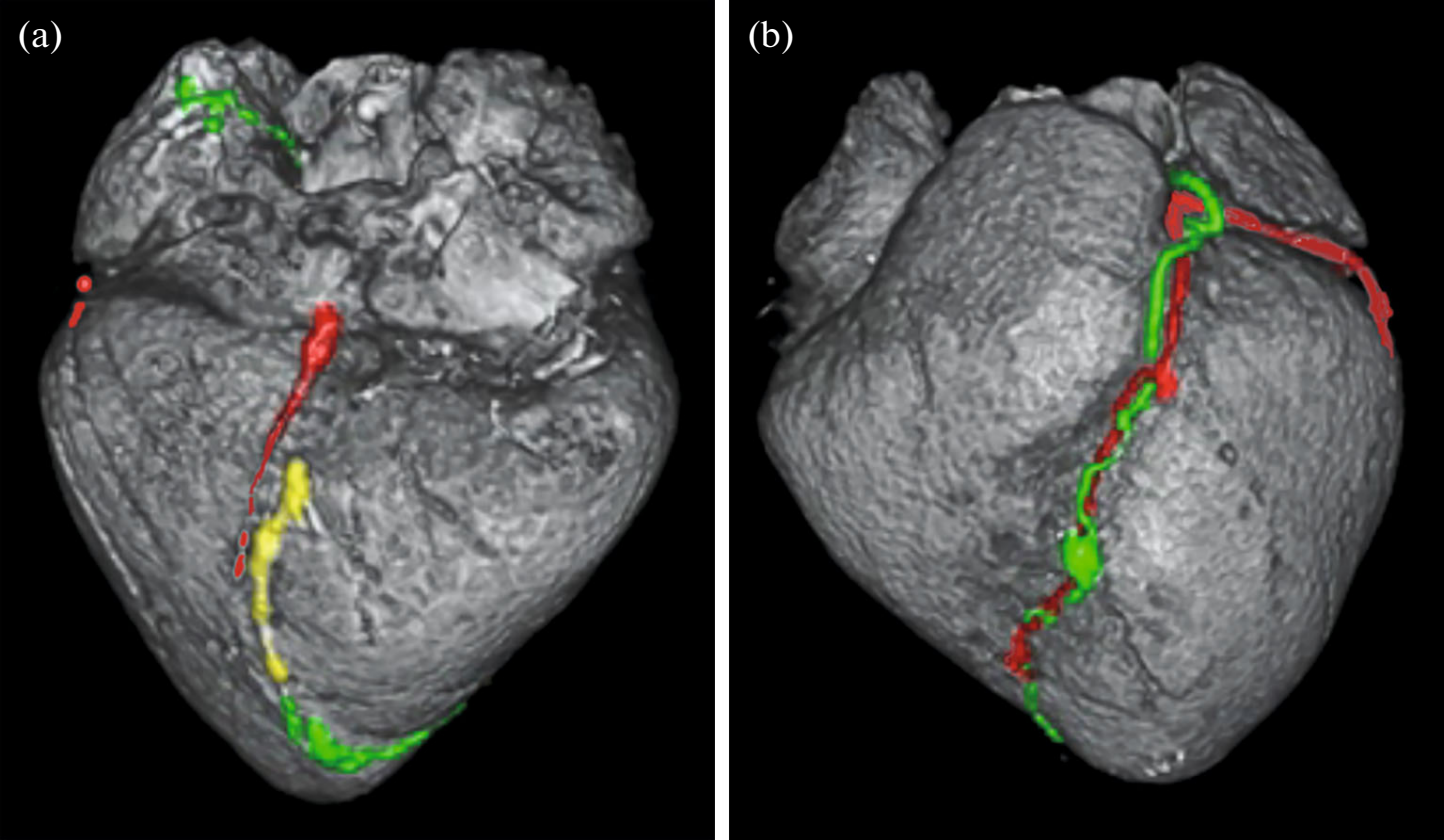
3D reconstructed CT image of the pig heart. (a) inferior wall of the heart, (b) anterior wall of the heart. The coronary arteries are shown in red, the anterior lymphatic collector (ALC) is shown in green, and the posterior lymphatic collector is shown in yellow. The beginning of the ALC on the inferior wall of the heart is seen.

In the distal third of the RCA and posterior interventricular artery, the lymph flow is directed toward the apex of the heart, in phase with the arterial flow, which was not noted in previously published studies. The lateral wall lymphatic collector was commonly (*n* = 16) seen as a bundle on the lateral wall, forming a relatively short trunk that drains into the interatrial groove and connects with the anterior trunk. CT scans demonstrate the connection between the coronary arteries, veins, and lymphatic vessels.

**Fig. 4.**
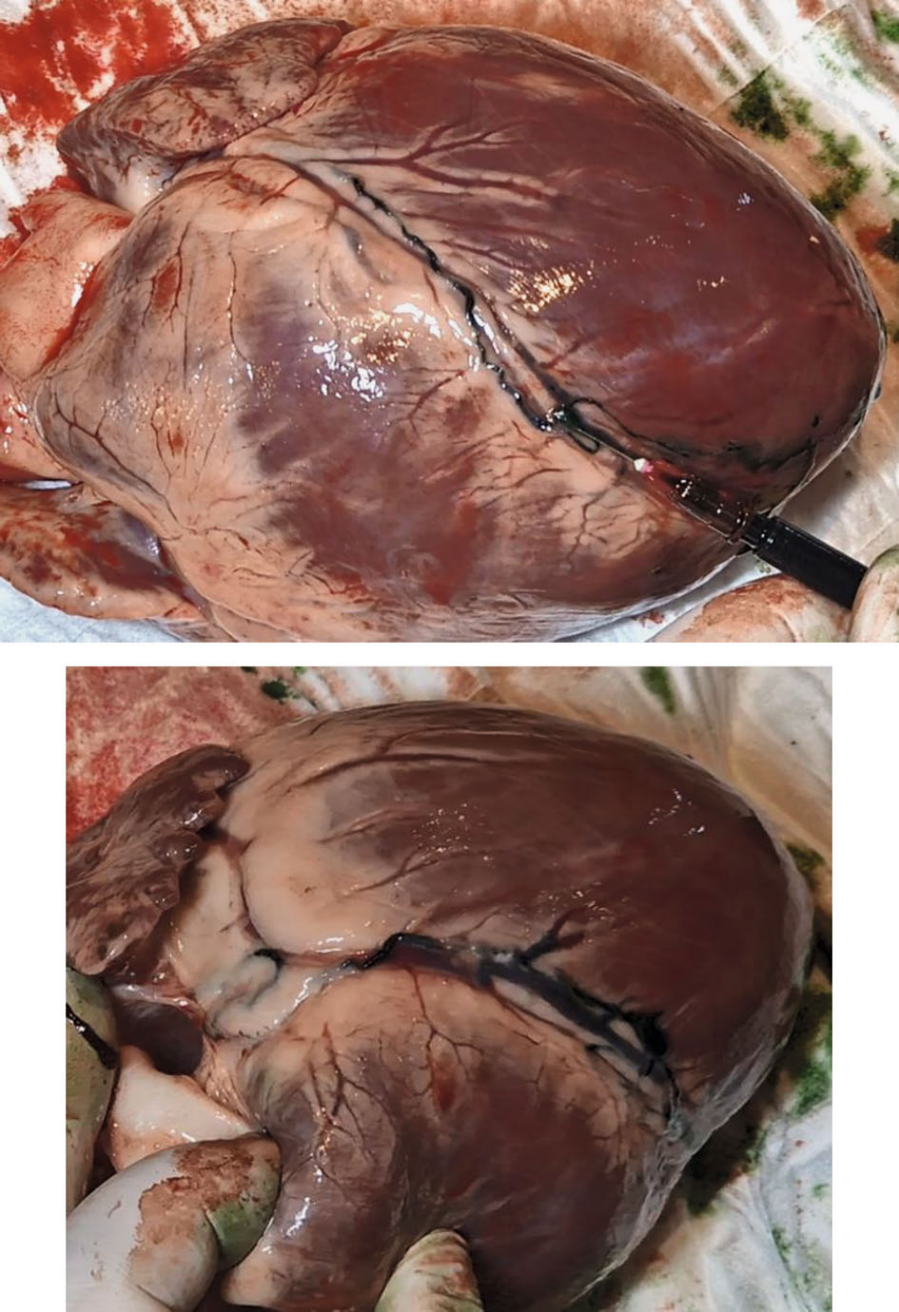
View of the pig heart after the injection of lymphatic vessels with the original dye, distal (left) and proximal (right) sections of the LCA, including the loop in the bifurcation area of the left coronary artery trunk.

## DISCUSSION

Although the role of the lymphatic system in the development of the atherosclerotic process has been studied for a long time, relatively few works have been devoted to it, and there is almost no practical application of the obtained data for patients with atherosclerosis. For example, in the literature there are various attempts to systematize the anatomy of the lymphatic system. Johnson and Blake in 1966 describe the paracoronary main vessels that form the lymphatic duct of the atrioventricular groove and, further, the main cardiac lymphatic duct [8]. In a later study by Eliška and Elisková (1980), the authors focused on the regional lymph flow of the cardiac conduction system, primarily at the microscopic level [7]. In the work by Riquet et al. (2000), attention was paid to the outflow of lymph from the heart to the regional lymph nodes; however, the intracardiac anatomy was not described in detail [6]. The structure of the cardiac lymphatic ducts was most fully described by Kappler et al., and we relied on the anatomical classification proposed by these authors [4]. However, the last work does not describe accurately the lymph flow direction in different areas of the myocardium. At the same time, it is known that the anatomy of the cardiac lymphatic system and lymph flow disorders may be important in explaining some aspects of atherosclerosis, as well as some differences in long-term outcomes after percutaneous coronary interventions and bypass grafting [10]. On the one hand, it was shown that the number of lymphatic vessels in the arterial wall is positively correlated with the intimal thickness (*r* = 0.37, *p* < 0.05) and the age of patients (*r* = 0.3, *p* < 0.05) [11]. On the other hand, in patients after mammary gland resection and lymph node dissection, the arterial wall thickness was significantly increased [3]. Both facts suggest that the enlargement of lymphatic vessels in the arterial wall occurs secondarily to the disruption of proximal lymph flow. In addition, a study of 51 patients with lower limb ischemia performed in 1996 showed that indirect lymphatic drainage improved the clinical picture in patients with stages IIb and IIIa ischemia [12]. In a study by Fujita, it was shown that, in case of extended stenting, a continuous stenting of the right coronary artery is necessary; otherwise, early restenosis develops precisely in the spaces between the stents [13]. It can be assumed that the disruption of the lymph flow in the segment not covered by stents may be one of the mechanisms leading to narrowing of the vessel in this zone. Furthermore, it has been shown that the incidence of MACCE is not the same after stenting different coronary arteries: an unfavourable outcome is almost twice as rare after PCI of the right coronary artery and more than three times as rare after stenting of the circumflex artery than after stenting of the anterior interventricular artery of the same diameter [14]. This phenomenon can largely be explained by the differences in the direction of lymphatic flow in the regions of these coronary vessels that we have identified. However, further experimental studies are required to confirm the contribution of lymph flow to the progression of atherosclerosis in different segments of the coronary vessels.

## CONCLUSIONS

The anterior lymphatic trunk has been shown to collect lymph from both the LAD and the apical part of the RCA region, which may explain the better outcome after RCA stenting compared to the circumflex artery or LAD due to the absence of lymph flow impairment. In addition, the new technique for visualizing lymphatic vessels (stepwise intramuscular injection of a fat-soluble dye followed by mixing it with lipiodol) allows improved and simplified visualization of both deep and superficial portions of lymphatic vessels running in the epicardial and mediastinal adipose tissue.
